# Lead Poisoning among Women

**Published:** 1900-07-07

**Authors:** 


### LEAD POISONING AMONG WOMEN.

The influence of lead is a factor in the production of disease which it is impossible to get away from. Not unnaturally it is far more widespread in some districts than in others. Where local industries involve the use of this metal or its compounds, as in the reduction of the ore, in the manufacture of white lead, in the grinding of paints, and in the glazing of pottery, all forms of lead poisoning, both acute and chronic, are constantly met with; while in places, the water supply of which is "soft," a more or less wide diffusion of a minor degree of chronic lead poisoning may complicate and aggravate maladies of all kinds. In other places, however, lead poisoning is hardly met with except in relation with certain trades and among people who are exposed to particular risks. It is well, then, to be reminded, as we are, by Dr. Ransom[1] of the extent to which diachylon, or oleate of lead, is used by women as an abortifacient, and of the frequency with which its use for this purpose leads to some of the most acute and serious forms of plumbism, for this is an insidious cause of the disease which, if one were not aware of its prevalence, one might easily overlook. Dr. Ransom gives cases in which severe lead encephalopathy was produced in this way, and mentions several in which other forms of lead poisoning were the result of the same practice, and says that there can be no doubt that diachylon is largely used by women of various classes to procure abortion. It is a substance which is easily purchased. Anyone can go into a chemist's shop and buy a pennyworth of diachylon without being asked any questions except as to whether the purchaser wants it spread or in the mass. Indeed, penny balls of the emplastrum plumbi are, it is said, kept, even by the most respectable chemists, ready wrapped in a handy drawer, and there is absolutely no restriction on its sale. No doubt the drug is an uncertain abortifacient, often failing in the purpose for which it is given. But it is a very sure producer of disease, always endangering, and often destroying life, or leaving permanent mental and bodily enfeeblement. It is then a very serious question whether something should not be done to prevent the indiscriminate sale of such a drug, one with which, for the expenditure of a penny, a woman may not only empty the uterus, but may cause grave, and not infrequently fatal, disease of the bowels, the kidneys, and the brain. The most striking and interesting point brought out by Dr. Ransom is the amount of disease which may be produced by a very short course of the diachylon, and the continued persistence of its deleterious and poisonous effects long after its use has been entirely discontinued. Of course, in this matter there were only the statements of the patients to go upon, but, so far as they went, they showed that in some cases quite a few doses, or even one dose, had produced long-lasting mischief.

[1] Brit. Med. Jour., June 30.

